# A Deep Learning-Enabled Skin-Inspired Pressure Sensor for Complicated Recognition Tasks with Ultralong Life

**DOI:** 10.34133/research.0157

**Published:** 2023-06-07

**Authors:** Yingxi Xie, Xiaohua Wu, Xiangbao Huang, Qinghua Liang, Shiping Deng, Zeji Wu, Yunpeng Yao, Longsheng Lu

**Affiliations:** School of Mechanical & Automotive Engineering, South China University of Technology, Guangzhou, China.

## Abstract

Flexible full-textile pressure sensor is able to integrate with clothing directly, which has drawn extensive attention from scholars recently. But the realization of flexible full-textile pressure sensor with high sensitivity, wide detection range, and long working life remains challenge. Complex recognition tasks necessitate intricate sensor arrays that require extensive data processing and are susceptible to damage. The human skin is capable of interpreting tactile signals, such as sliding, by encoding pressure changes and performing complex perceptual tasks. Inspired by the skin, we have developed a simple dip-and-dry approach to fabricate a full-textile pressure sensor with signal transmission layers, protective layers, and sensing layers. The sensor achieves high sensitivity (2.16 kPa^−1^), ultrawide detection range (0 to 155.485 kPa), impressive mechanical stability of 1 million loading/unloading cycles without fatigue, and low material cost. The signal transmission layers that collect local signals enable real-world complicated task recognition through one single sensor. We developed an artificial Internet of Things system utilizing a single sensor, which successfully achieved high accuracy in 4 tasks, including handwriting digit recognition and human activity recognition. The results demonstrate that skin-inspired full-textile sensor paves a promising route toward the development of electronic textiles with important potential in real-world applications, including human–machine interaction and human activity detection.

## Introduction

In recent years, with the rapid development of artificial Internet of Things (AIoT) and artificial intelligence technology, more and more flexible wearable devices can be competent for complicated recognition tasks, leading to a progressive revolution [1–3]. Among them, electronic textiles (e-textiles) that integrate various electronic devices such as flexible sensors [4], energy collectors [5], and energy storage devices [6] into fabrics have been considered as one kind of the most ideal flexible wearable devices and attracted tremendous attention of many researchers worldwide [7,8]. But the fatigue research of the current flexible pressure sensor is mostly stuck in the low cycle (10^4^ to 10^5^) [9,10], and the required high cycle fatigue performance of the sensor is rarely studied in more scenarios. To ensure good health, it is recommended that an average person takes about 3.5 million steps a year [11]. Therefore, flexible pressure sensors capable of detecting human walking muscle with a lifespan exceeding 1 million cycles are highly desirable. Fabric fibers can prevent catastrophic crack propagation of the structure and have high bending fatigue resistance, which is higher than that of congeneric products based on flexible films [12]. Compared to flexible polymer film electronic devices, electronic fabrics are more comfortable and breathable, which receive considerable attentions in various applications [13,14], including sports [4,15], robot perception [16], human–machine interaction [17], and healthcare [18]. Recently, researchers have fabricated many textile-based pressure sensors by using laser-scribing [19], carbonization [20], chemical synthesis [21,22], and so on. However, the current manufacture process of full-textile pressure sensors is laborious and expensive. Moreover, the substrates, such as ion–gel film [23], polydimethylsiloxane (PDMS) [24], and polyimide (PI), for the manufacture process of full-textile pressure sensors are not favorable for e-textiles. Therefore, to meet the needs of full-range pressure of application scenarios such as human motion detection and human–machine interaction, full-textile pressure sensor with high sensitivity, large detection range, and long working life are still in urgent need.

In addition to the requirements for high-performance sensors, another new trend is to combine e-textiles with smart modules to realize healthcare, smart home, and other smart applications [25]. For example, in human–machine interaction applications, researchers have demonstrated the ability of pressure sensor arrays fabricated by inkjet printing [26–29] to recognize spatial and temporal signals, such as handwritten digit [30,31], Braille [32], and different sentences in multiple languages [33,34]. Complicated recognition tasks necessitate a high-density flexible pressure sensor array in a specific area. The denser the array, the greater the capacity to detect subtle and intricate actions, but also the more fragile the sensor devices, the more complex the circuit design, the more voluminous the data to be collected, and the more intricate the signal analysis. Some researchers have combined artificial intelligence to achieve multiplayer handwriting recognition using only one sensor instead of array [34,35]. Nevertheless, few researchers have studied the performance using one sensor on real-world complex tasks.

The dermis of human skin contains receptors that detect mechanical signals, and these signals are processed by nerve cells and transmitted to the brain to perform complex tasks. Inspired by the skin, we have developed a graphene-coated full-textile (Gft) pressure sensor with signal transmission layers, protective layers, and sensing layers by a simple dip-and-dry approach. It is equipped with high sensitivity, wide monitoring range, and superior long-term stability and can be used for human–machine interaction and human activity monitoring wearable devices. Because of the simple preparation method of dip drying and the avoidance of complex high temperature or reduction methods to prepare graphene, the Gft sensor can be automatically manufactured with high consistency performance by modern textile processes. The signal transmission layer proposed can efficiently gather faint signals locally, thereby achieving functionalities similar to those of flexible pressure sensing arrays. In addition, the sensor exhibits superior mechanical properties and more dependable electrical connections than pressure arrays. We built an AIoT system based on a Gft sensor and verified its performance in human activity monitoring and human–machine interaction scenarios. High accuracy was achieved on the 2 human activity recognition datasets and the 2 handwritten digit recognition datasets collected. As a proof of concept for the smart home, the trained handwritten digit recognition model was deployed on the AIoT system to detect the collected signals in real time through cloud computing. This work provides another promising strategy for achieving complicated recognition tasks.

## Results

### Skin-inspired Gft sensor principal design and manufacturing

**Fig. 1.**
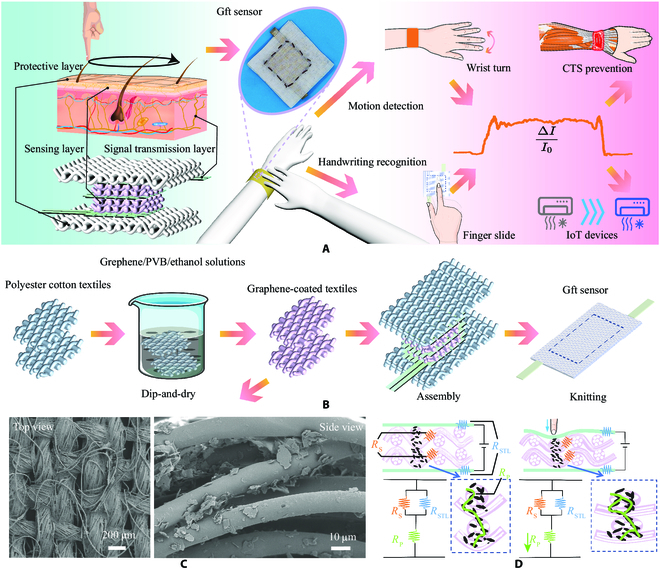
Sensing principle and practical applications. (A) Structure and artificial intelligence applications of the skin-inspired Gft sensor. (B) Fabrication and assembly processes of the skin-inspired Gft sensor. (C) Scanning electron microscopy images of the top and side sections of the 4 wt% graphene/PVB-coated conductive fabric. (D) Analysis of the piezoresistive effect of the Gft sensor.

Human skin is full of the ingenious design of nature. It can collect mechanical signals from local locations on the skin through the pervasive nerves. For example, when recognizing handwritten digits on the skin, the brain can deduce the character by combining signal changes in time and space. As shown in Fig. 1A, the skin-inspired Gft sensor has a multilayer structure with protective layers, sensing layers, and signal transmission layers. The protective layer is composed of 2 layers of clean polyester–cotton textiles that are stacked to immobilize and isolate the inner layers. It functions similarly to the insulating stratum corneum located on the surface of the human body, which can receive pressure input while effectively blocking external interference to prevent disturbance of electrical signals in the nerves. The sensing layers act like tissues and receptors on the skin that process mechanical input, consisting of 4 layers of graphene/polyvinyl butyral (PVB)-coated polyester–cotton textiles. Its woven structure and multilayer were pressure sensitive, resulting in piezoresistive signals. Figure 1C further demonstrates this point of view. Microscopically, graphene adheres tightly to the polyester–cotton fibers, forming a polyester–cotton graphene sheet bond. Graphene flakes attached to the fiber surface randomly overlap and stack with each other to achieve macroscopic conductivity. At the same time, during the contact of different layers of conductive fabrics, more graphene flakes are squeezed to establish electrical connections, realizing the overall electrical conductivity change. But in some regions with higher graphene concentrations, graphene deposits have been formed, which are unstable and prone to collapse under pressure. The signal transmission layers were composed of copper–nickel polyester fabric with good electrical conductivity, which was interwoven with the sensing layer to collect locally generated piezoresistive signals and act as ubiquitous nerves on the skin (see Materials and Methods for detailed preparation and assembly procedures.)

As shown in Fig. 1B, the polyester–cotton textile is covered with a conductive layer of graphene/PVB through dip coating, drying, and cleaning to achieve good conductivity. Then, the protective layer, signal transmission layer, and sensing layer were sewn together to form the integrated sensor. As shown in Table S1, the Gft sensor shows ultralow manufacturing costs (a thousand sensors for only 1.0885 dollars) and very strong adaptability to the modern textile industry, which can easily achieve large-scale production with industrialization and consistent performance. As shown in Fig. S1, the average thickness of the Gft sensor is 1.6274 mm. Because sewing during the integration process introduced a certain pre-pressure to the Gft sensor, the integrated thickness was less than the sum of the thicknesses of the layers. As shown in Fig. 1D, when subjected to lower pressures, uniform contact points and conductive paths are established between graphene/PVB-coated polyester–cotton textiles. As the pressure increases, slippage occurs between different conductive textiles, and more contact points appear between the polyester–cotton fiber, which can further reduce resistance. The neural-like signal transmission layer plays a great role in the sensing of local signals. When the local signal is depressed, the sensitivity of the simplified model is shown in Eqs. 1 and 2:

$$S_{\mathrm{skin}-\mathrm{like}}=\frac{\frac{R_{S}R_{\mathrm{STL}}}{R_{S}+R_{\mathrm{STL}}}+R_{F}}{\frac{R_{S}R_{\mathrm{STL}}}{R_{S}+R_{\mathrm{STL}}}+{R_{F}}^{\prime }}-1$$ (1)

$$S_{\mathrm{without}\;R_{\mathrm{STL}}}=\frac{R_{S}+R_{F}}{R_{S}+{R_{F}}^{\prime }}-1$$ (2)

where *S*_skin − like_ represents the local sensitivity of the Gft sensor and *S*_without *R*_STL__ represents the local sensitivity of the Gft sensor without the signal transmission layers. *R*_S_, *R*_STL_, *R*_F_, and *R*_F_^′^ represent the transmission resistance of sensing layers, the resistance of the signal transmission layer, the resistance conductive paths formed by the sensing layer, and the changed *R*_F_ under increasing pressure, respectively. Here, we assume that the contact resistance between the transport layers in the 2 simplified models is consistent and is connected in series to the conductive path. Because the signal transport layer has good conductivity, the influence of the contact impedance between the sensing layer and the signal transport layer on the simplified model is ignored. Comparing Eqs. 1 and 2, it is observed that the skin-inspired Gft sensor with the signal transmission layer has higher sensitivity.

### Gft sensor performance characterization

**Fig. 2.**
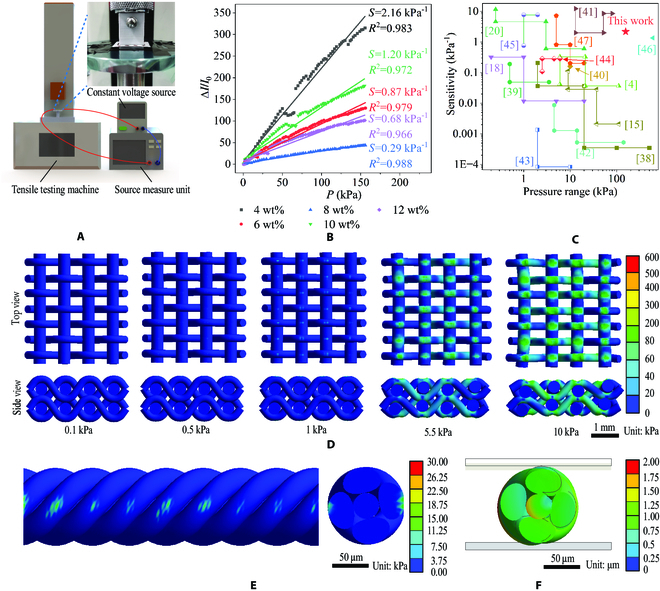
Performance characterization of the Gft sensor. (A) Schematic diagram of an experimental device for tensile testing of the Gft sensor. (B) Performance comparison of Gft sensors manufactured by dip coating with different graphene concentrations. (C) Performance comparison between the Gft sensor and the reported full-textile pressure sensor. (D) Stress distribution of the sensing layer under different pressures. (E) Stress distribution and (F) deformation of a single fiber under compression (zoom in 5 times for display).

As shown in Fig. 2A, the Gft sensor is fixed on the tensile testing machine by insulating strips and was connected in series with the direct current (DC) constant voltage source and source measuring unit via wires to measure the current change of the Gft sensor during the pressure process. As shown in Fig. S2F, 4, 6, 8, 10, and 12 wt% graphene concentrations are studied for their dip coating effects, and the conductive textiles manufactured with 4 wt% graphene concentration had the smallest mass loss during cleaning (0.4 g). The results show that the graphene material in the Gft sensor exhibits delamination tendencies under various extreme conditions. However, since the dense signal transmission layer and the protective layer effectively isolate the fall particles, there are no concerns about allergies or safety effects. Compared to Gft sensors of other concentrations, the 4 wt% Gft sensor has better sensitivity due to the uniform adhesion of graphene to polyester–cotton and the less accumulation of graphene. The results show that pressure sensitivities of 4, 6, 8, 10, and 12 wt% were 2.16, 0.87, 0.29, 1.20, and 0.68 kPa^−1^, respectively (Fig. 2B). The lower-concentration Gft sensor achieved a higher linearity (>0.979) in a large detection range (0 to 155.485 kPa), and the linearity showed a decreasing trend when the concentration was greater than 8 wt%. The reasons for the gradual decreased in sensitivity mainly include the following: (a) An increase in graphene content results in more interstices of the fabric being filled and covered with graphene, which reduces the number of mechanically sensitive structures in the sensing layer (when the textile's structure is completely covered with graphene, the Gft sensor prototype will degrade into a pressure sensor composed of multiple layers) and (b) an increase in graphene content leads to a higher initial current and a smaller proportion of the changeable current, making it harder to detect a corresponding change in current caused by subsequent pressure. Conversely, an increase in graphene/PVB concentration leads to a lower Young modulus of the original textile [36], resulting in a larger response current. This is the primary reason for the Gft sensor's increased sensitivity following an increase in graphene concentration. Under normal circumstances, the pressure generated by the daily movement of the human body is within 0 to 100 kPa [37], so the detection range of the measured Gft sensor is suitable for detecting the daily movement of the human body. As shown in Fig. 2B, the current response of the Gft sensor decreases by about 50 kPa. The decreasing might be caused by embedding of braided fibers at a certain critical force. But after exceeding this range of forces, the Gft sensor's response was prepared by the slip of fine fibers, returning to the original sensitivity and linearity. To quantitatively analyze the sensitive performance of the Gft sensor, the sensitivity and pressure range were compared with those of previously reported full-textile flexible pressure sensors (Fig. 2C) [4,15,18,20,38–47]. It is observed that the Gft sensor achieves a relatively high sensitivity (2.16 kPa^−1^) and a large detection range (0 to 155.485 kPa).

**Fig. 3.**
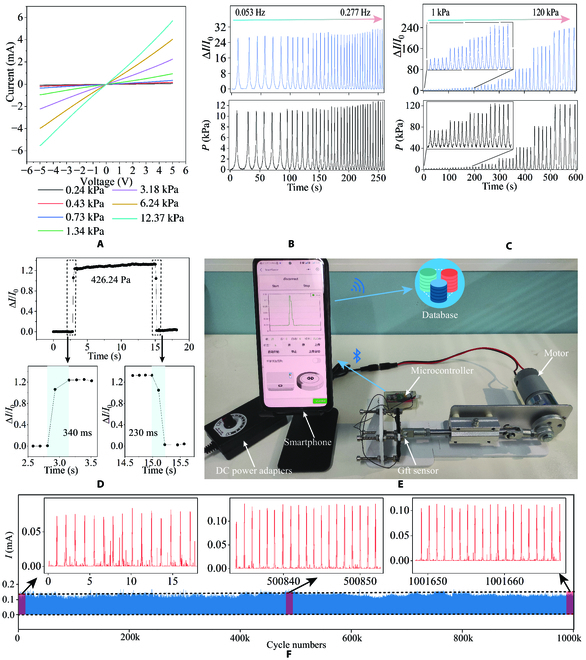
Sensor actual use performance test. (A) Current–voltage curves of the Gft sensor under various pressures. (B) Current response of the Gft sensor at an external force of 10 kPa; the frequency increases from 0.053 to 0.277 Hz. (C) The current response of the Gft sensor under external force increases from 1 to 120 kPa. (D) Response and recovery time of the Gft sensor under the pressure of 426.24 Pa. (E) Wireless cyclic sensing performance test system. (F) Stability and reliability of the Gft sensor over 1 million cycles.

To further study the piezoresistive mechanical behavior of the sensing layer, a simplified finite element model with only a few nodes was established (Fig. S3A). The fiber diameter of the sensing layer was measured and used to build proportional finite element model (Fig. S2). The stress distribution and variation trend of the sensing layer were studied using 2 flat pressure plates. The results show that the established sensing layer model increases linearly with the increase of external force (Fig. S3B and C), explaining the linear response of the Gft sensor in the full pressure range. As shown in Fig. 3D, the stress is mainly concentrated in the fibers above and below the nodes of the woven structure. The fibers constructed in the finite element method (FEM) model were woven from finer polyester–cotton fibers (Fig. 2E and F), and there will be relative movement between fibers before the model crushes at 10 kPa or more, to have a larger pressure bearing range (Fig. 3E and F).

The pressure sensing performance of the Gft sensor was systematically characterized. Figure 3A shows the current–voltage curves of the Gft sensor under different external pressures. It is observed that the Gft sensor provides good linear ohmic behavior in a large voltage range (from −5 V to 5 V) when the applied pressure increases from 240 Pa to 12.37 kPa, showing the perfect conductive networks of the sensing layers and signal transmission layers. As shown in Fig. 3B and C, the Gft sensor keeps the constant current at an increasing frequency (from 0.053 to 0.277 Hz) and increasing pressures (from 1 to 120 kPa). The slight difference was caused by the overshoot of the tensile testing machine at a higher speed, which can be observed on the pressure curves. In addition, the increasing current with increasing pressure at Fig. 3C is also consistent with the results in Fig. 2B. As shown in Fig. 3D, the Gft sensor showed a rapid response (340 ms) and recovery time (230 ms) under the pressure of 426.24 Pa, which has great superiority for real-time human motion monitoring and human–machine interaction. The Gft sensor was fixed to the cycle test device shown in Fig. 3E. The motor was powered by the DC power adapter, which drove the gear and cranks linkage mechanism to press the Gft sensor for a fixed displacement. The pressure end is similar to the area of the human finger, and the pressure is pressed in a fixed position for the Gft sensor fatigue test of the press–release cycle. The resulting current (~ 0.1 mA) was close to the peak current applied to handwritten digits subsequently. The microcontroller used the analog-to-digital converter (ADC) to collect current from the Gft sensor in real time and Bluetooth module to transmit data to the applet on the mobile phone. The applet collected and uploaded current data to the cloud database. Figure 3F illustrates the repeatability and stability of the Gft sensor under over 1 million loading/unloading cycles. Regardless of changes in mechanical lubrication conditions and deterioration in sensor installation conditions, the reason for the small variation in Gft sensor response in 1 million cycles was likely caused by the shedding of few graphene with poor bonding force on the sensing layer. The response of the Gft sensor was noisier in the first 15 cycles than in the last 15 cycles of 1 million, which indicated that graphene shedding occurs during the initial use of the Gft sensor. According to the Basquin’s equation [48,49] of the high cycle fatigue, the working life (*N_f_*) is negatively logarithmically related to the stress (∆σ*_a_*) inside the Gft sensor at each load:

$$\frac{\Delta \sigma_{a}}{2}=\sigma_{f}^{\prime }{\left(2N_{f}\right)}^{b}$$ (3)

where $\sigma_{f}^{\prime }$ is the fatigue strength coefficient and *b* is the fatigue strength exponent. As shown in Fig. S4A, the Gft sensor with the signal transmission layer exhibits a smaller stress concentration after compression. According to Eq. 3 [48,49], the Gft sensor has a longer working life due to the presence of the signal transmission layer. The long-term continuous measurement demonstrates the ultralong working life and stable performance of the Gft sensor and built AIoT system.

### Application of Gft sensor in actual tasks

**Fig. 4.**
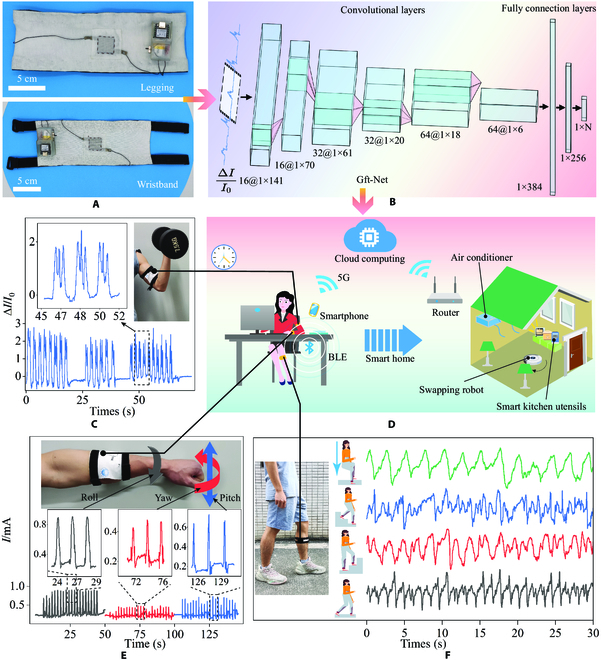
(A) Photograph of the assembled smart legging and wristband. (B) The established CNNs are used for human activity recognition and handwritten digit recognition (N is the class number of tasks, 16@1×141 means 16 channels and each channel is a matrix of size 1 × 141.). Real-time sensing signal recording of the Gft sensor for human activity recognition, including (C) biceps contraction and relaxation, (E) wrist rotation detection, and (F) calf muscle changes during daily squatting, going downstairs, going upstairs, and walking. (D) The built-in real-time data uploaded to the cloud, cloud storage, and computing, signal recognition system, and Gft sensor human–machine interaction realize smart home design.

Due to the ingenious skin-inspired structure of the Gft sensor, the local pressure distributed in the sensor can be collected by the signal transmission layer to integrate into the current response. Human activities and handwritten digits can be recognized by combining with artificial intelligence algorithms. The Gft sensors were sewn onto a legging and wristband (Fig. 4A). The current response of the Gft sensor was collected with a microcontroller at a frequency of 75 Hz and transmitted to the smartphone by Bluetooth low energy (BLE). After the applet on the smartphone established a connection with the microcontroller, the pressure sensor data were collected and sent to the cloud database. As shown in Fig. 4B, a convolutional neural network (CNN) for analyzing the current signal of the sensor and pattern recognition (named Gft-net) is built. The convolutional layers are used to process and extract features from the input signal, and fully connection layers are used to combine features and output classification results based on features (detailed Gft-net parameters are shown in Table S3). In addition, the trained Gft-net was deployed on the Tencent CloudBase Run platform and realized handwritten digit recognition in real time (Fig. 4D).

To demonstrate its application prospects, a Gft sensor was installed on the biceps to detect variations in muscle movement during dumbbell exercises. As shown in Fig. 4C, when the arm lifts a dumbbell and makes the lifting and lowering movements, the Gft sensor can accurately collect stable and repeatable sensing signals. There were characteristic peaks that can be discerned in the response current curve, showing a good ability to distinguish subtle movements of the biceps brachii. Similarly, the wristband on the wrist detected rotation signals in 3 directions: wrist–roll, wrist–pitch, and wrist–yaw. As shown in Fig. 4E, the response current of the Gft sensor under the movement of the 3 directions shows different peak current response characteristics. Wrist heath monitoring can be achieved through rotation mode and distribution, and frequency count in daily life. This is of great significance to many workers who require frequent repetitive wrist efforts and are often plagued by carpal tunnel syndrome (CTS). The emergence of Gft sensors makes it possible to monitor the health of their wrists and remind them of their daily activities to prevent CTS.

Leggings were worn on the lower leg to collect Gft sensor current response curves during squatting, going downstairs, going upstairs, and walking. As shown in Fig. 4F, the current of the Gft sensor changes periodically with repeated human movements. The current response corresponding to each action has different characteristic peaks, among which walking and squatting have the most repetitive curves. However, due to the complex stress conditions during the movement of the upstairs and downstairs, the curves have more noise, which will hinder signal extraction. As shown in Fig. S5, the chaotic upper and lower floor data are performed by fast Fourier transform (FFT), and a clearer and more consistent characteristic peak is obtained after a 3-Hz low-pass filter. The Gft sensor can easily collect signal differences on human activities, showing potential applications in guiding physical training and muscle injury recovery by detecting human movements.

**Fig. 5.**
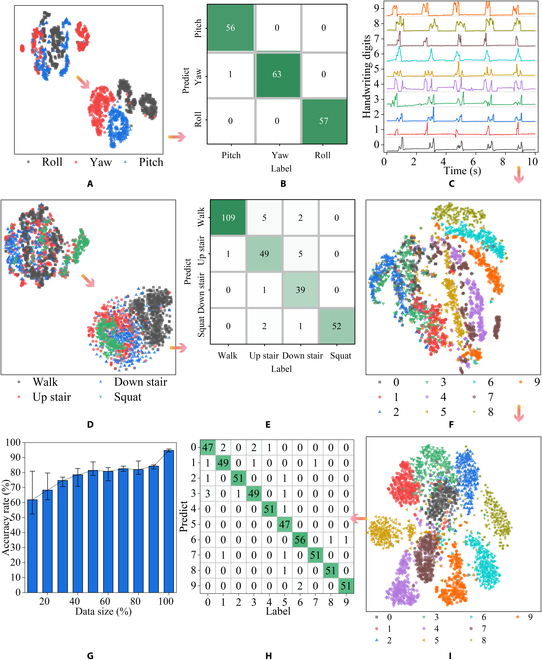
Pattern recognition for complex tasks of sensors. (A) Dimension reduction visualization of the collected wrist rotation dataset and the features extracted by CNN t-SNE data. (B) Confusion matrix of the recognition results of wrist turn test set. (C) The 10-s response current curves of different handwritten digits. (D) t-SNE dimension reduction visualization of human activity dataset and its features. (E) Confusion matrix of the recognition results of human activity test set. (F) t-SNE dimension reduction output of handwritten digits on the wristband dataset. (G) Graph of recognition accuracy as training data increase. (H) Confusion matrix of the recognition results of the handwritten digit test set. (I) t-SNE dimension reduction visualization of the features of the handwritten digit dataset.

To investigate the effectiveness and accuracy of Gft sensor extracting target information feature, we use deep learning algorithms. It is used for effective data processing to ensure that sensor signal can be fully resolved. The 2-s data were collected, segmented, and uploaded to the cloud database by the microcontroller at 75 Hz. We use the t-distributed random neighbor embedding (t-SNE) algorithm to reduce high-dimensional data to 2-dimensional data. Seven hundred seven data segments were collected as training dataset for wrist rotation recognition, and 177 data were used to test the accuracy of the model. The distribution of all datasets was obtained by using t-SNE dimensionality reduction. As shown in Fig. 5A, it can be observed that the 3 signals are grouped into multiple clusters, which can be easily distinguished. The feature outputs by the trained CNN were also dimensioned by the t-SNE algorithm, and the resulting distribution map presents clearer classification decision boundaries, which allows the fully connected layer to easily combine features and classify the 3 rotations. Typical confusion matrix shown in Fig. 5B further supports this conclusion, with 99.44% accuracy in the test set. These results not only demonstrate the high quality of the pressure signal data collected by the Gft sensor but also confirm the great potential of AIoT that combines sensing and recognition.

To further demonstrate the great potential of sensors in human activity monitoring tasks, we established a training dataset for the 4 activity conditions detected in Fig. 4F, including 1,060 data, to train a 4-classification network. An additional 266 data were collected to test the accuracy of the trained network. The distribution of the collected data after dimensionality reduction is shown in Fig. 5D. Due to the increased complexity of the signal, some signals of different types were mixed. The features extracted by the trained CNN were closer to data from the wrist rotation dataset, but there were still clear decision boundaries. Figure 5E further demonstrates this conclusion, with the test set achieving an accuracy rate of 93.61%. As shown in Fig. 4F, the pressure conditions for going upstairs and going downstairs were more complex; there was more noise, so these 2 types of data were more likely to be misclassified. The convolutional layers learned the eigenvalues of the filter over a large amount of data and extracted accurate features. The fully connected layers accurately identified the behavior of going upstairs and downstairs by combining features.

In addition to the tasks of human activity, we also verified the vast potential of the Gft sensor in human–machine order recognition. Human skin interprets sliding signals on the skin by remembering and feeling changes in pressure. Due to the clever design of the signal transmission layers of the Gft sensor, local signals can be easily collected, so the recognition of handwritten symbols on the Gft sensor can be realized. Here, we select 10 Arabic numerals as sensor detection targets. By writing on the wristband worn on the wrist, 2,626 data were collected and randomly divided into training datasets (2,100 data) and test sets (526 data) by category. As shown in Fig. 5E, we display a waveform comparison diagram of handwritten digits within 10s. The signal waveform of the same data is highly reproducible, and each datum has different waveform details. This proves that the Gft sensor's unique structure can collect the pressure change of each local area of the sensor to achieve an efficient collection of handwritten data signals. Due to the large difference in waveforms of handwritten digital signals, the data distribution obtained after t-SNE dimensionality reduction has a higher concentration, and the different numbers are far apart (Fig. 5F). The trained CNN can extract more differentiated features for subsequent classification (Fig. 5I).

As shown in Fig. 5H, the classification results have a higher accuracy and recall rate, and the accuracy rate in the test set reaches 95.63% (Table). Figure 5G shows the effect of the amount of data collected on model accuracy. When 90% of the training dataset data was added to the training, the average accuracy of the 5-fold cross-trained model gradually increased from 61.90% to 95.63%. This indicates that when dealing with complex recognition tasks, increasing the volume of data can perform the recognition. It further demonstrates the usability of the Gft sensor in more complex applications. To prove the usability of the Gft sensor in handwritten digits for human–machine interaction on other sides, we collected another handwritten digit dataset on a glass plate for training and achieved an accuracy rate of 96.61%. We deployed the trained model on the Tencent CloudBase Run platform (see Materials and Methods) and used it for real-world recognition tests (Fig. S6). The smartphone recognizes handwritten digits and simulates turning on or off smart home devices through preset rules. The above results show that the skin-inspired Gft sensor can be highly integrated with artificial intelligence and intelligent IoT technologies to achieve complex tasks such as human activity recognition and human–machine interaction.

**Table.** The Gft sensor datasets of different tasks.

Dataset	Wrist turn	Human activities	Handwriting digits on the wristband	Handwriting digits on a glass plate
Training set size	707	1,060	2,100	2,121
Test set size	177	266	526	531
Class numbers	3	4	10	10
Mean current	0.040	0.072	0.021	0.017
Standard deviation	0.016	0.014	0.011	0.010
Accuracy	99.44%	93.61%	95.63%	96.61%

## Discussion

The skin-inspired Gft sensor was prepared by interspersing a graphene/PVB/polyester–cotton fabric sensor with a copper–nickel conductive cloth. Due to the excellent flexibility and weaving structure of polyester–cotton and copper–nickel-plated fabrics, the Gft sensor showed high sensitivity (2.16 kPa^−1^), wide detection range (0 to 155.485 kPa), and long working life (>1 million cycles without fatigue), which is an urgent requirement for flexible textile-based pressure sensors. In addition, the signal transmission layer was designed to achieve lossless acquisition of response signals caused by local strain. The skin-inspired Gft sensor proposed by us can provide a new route toward pressure sensor array with high durability for promising applications in telemedicine and wearable devices. The data collected by the Gft sensor has a higher dimensional coupling, which provides valuable and challenging new application scenarios for data mining and pattern recognition. Future research directions include sensor stability, signal analysis algorithms based on few-shot learning or meta-learning, and lightweight models and deployment methods.

## Materials and Methods

### Fabrication of Gft sensor

First, polyester–cotton textiles (Dongguan Huamei buyi Co. Ltd., China) were cut into 20 × 20 mm^2^ and 40 × 40 mm^2^ by CO_2_ laser cutting system (Han's Laser Technology Industry Group Co. Ltd., China). Second, 100 mg of PVB (98 wt%, Sigma-Aldrich) powder was poured into 100 ml of ethanol and dissolved at 300 rpm magnetic rotation for 3 h to make a 1 g/l PVB ethanol solution. Third, 2 g of multilayer graphene powder (Suzhou Carbon Fung Graphene Technology Co. Ltd., China) was added to 48 g of the PVB/ethanol solution and sonicated to prepare a 4 wt% concentration of graphene/PVB ethanol solution. Then, the 20 × 20 mm^2^ polyester–cotton was immersed in the graphene/PVB ethanol solution for 5 min, followed by a drying process in the oven at 60 °C to dry. After 2 dip coating and drying processes, the conductive fabric was placed in deionized water at 200 rpm for 5 min and dried to obtain the final conductive fabric.

The copper–nickel-coated polyester fabric (Chenxi Technology Electronic Application Co. Ltd.) was laser cut into the shapes shown in Fig. S2E. Two 40 × 40 mm^2^ polyester cotton fabrics, four 20 × 20 mm^2^ conductive fabrics, and 3 copper–nickel-coated polyester fabrics were integrated into the assembly method shown in Fig. 1B and sewn into a pressure sensor by hand. The full-textile pressure sensor was soldered with 2 wires for subsequent testing and applications.

### FEM simulation setting

As shown in Fig. S3, a simplified finite element simulation model of the sensing layer was established based on the thickness and spacing of the actual polyester–cotton textile yarn. The density of polyester–cotton was set to 1.97 mg/m [50], and the Poisson ratio was set to 0.40 [51]. Assuming that the sensing layer has the same mechanical properties, a portion of it (4 × 5 nodes) was selected for finite element analysis. Remote constraints were set on all sides to limit the 5 degrees of freedom, and upper and lower plates were established to simulate the pressure. In addition, the deformation and stress distribution of a single fiber under compression are also simulated by finite element, and the diameter of the fine fiber was 35 μm. The external force experienced was 3 × 10^−6^ N pressurized by the upper and lower plates. Similarly, the pressure form of the Gft sensor with or without the signal transmission layer was also simulated. The stress distribution of the Gft sensor was simulated by applying a force of 1 N to the established FEA model for cyclic testing.

### Characterization

The microscopic morphologies were characterized by field emission scanning electron microscopy (Carl Zeiss) with a working distance of 9.2 mm and a high electron tension of 5 kV. The optical microscopic morphology of polyester–cotton textiles was observed by an autofocus measuring camera (Shenzhen Sangnond Instruments Co. Ltd.).

The Gft sensor was fixed on the compression platform of the tensile testing machine (3344L3927 2 kN, Instron) by tape during the test, and the indenter of the tensile testing machine is fixed with a 20 × 20 mm^2^ plane 3D printed with polylactic acid for applying uniform pressure to the sensor. During the performance characterization of pressure sensing, the source measurement unit (2400A, Keithley) and the constant voltage power supply with 1-V output were connected in series to measure the current response. The Gft sensor sensitivity is defined as Eq. 4 [52]:

$$S=\frac{\partial \frac{\Delta I}{I_{0}}}{\partial P}$$ (4)

In the performance test of different forces, frequencies, and transient responses, the LCR tester (TH2838, Jiangsu Changzhou Tonghui Electronics Company Ltd., Jiangsu, China) was used to measure the sensor response in real time. The sampling frequency of 1 kHz and the voltage of 1 V were internally selected to measure resistance and calculate current.

The thickness of the textiles and Gft sensor were measured by a thickness meter (International Material Tester, Dongguan) according to ISO 534. The contact pressure was 2 kPa, the measurement environment was 25 °C, and the humidity was <85%.

### Construction and composition of cyclic test system

As shown in Fig. 3E, the Gft sensor is fixed on a polylactic acid (PLA) plane whose spacing could be adjusted by 4 bolts, the mini telescopic machine (Shenzhen Shengda Co. Ltd.) was fixed at a horizontal distance of 19.9 cm from the sensor, and the telescopic machine speed was set to 30 rpm. The farthest end is 20.0 cm away from the center of the electrode, and the thrust is 18 N. At the same time, the real-time current response of the sensor was transmitted by the microcontroller to the cloud server.

### Sensor microcontroller system and AIoT system construction

The Arduino Seeed Sensor XIAO (Seeed Technology Co. Ltd.) microcontroller obtained a stable power supply by connecting a lithium battery with a rated voltage of 3.7 V. The sensor and a 10-kΩ sampling resistor are connected in series to 3.3 V and then connected to the Arduino analog input port to measure the midpoint analog voltage. The conversion relationship between the output value of the microcontroller and the actual current passing through the sensor is:

$$I_{\mathrm{Gft}}=0.33\times \frac{1024-\mathrm{output}}{1024}\mathrm{mA}$$ (5)

The internal program of the microcontroller was set to collect an analog voltage every 10 μs after receiving the instruction and publish it as a notify characteristic value of the BLE 4.0 service. The applet on the smartphone read the published analog voltage, calculated its corresponding current value according to Eq. 5, and uploaded the data at a frequency of 0.5 Hz. In the AIoT system, the smartphone applet could turn on the instruction recognition mode and transmit the 2-s signal to the service deployed in the Tencent CloudBase Run using the python flask library, which is called Gft-net under the PyTorch framework, to classify the input signal and return the recognition category and confidence in real time.

### Deep learning model training and evaluation

All training and test data come from the AIoT system built above, and the data length of the input Gft-net was (1,150). The network structure is shown in Fig. 4B and Table S3. The Gft-net network architecture used was consistent, and only the hyperparameters used for training and the number of output nodes of the fully connected layer were different. The training dataset and test set were randomly divided from the total dataset into a ratio of 8:2 without repeating, and the test data did not participate in the training and layer descent process during the training process.

## Data Availability

All data are available in the main text or the Supplementary Materials. The required data can be obtained from the corresponding author or coauthor.
