# Supplementary material for: A Deep Learning-Enabled Skin-Inspired Pressure Sensor for Complicated Recognition Tasks with Ultralong Life
Source: Research (Wash D C). 2023 Jun 7;6:0157. doi: 10.34133/research.0157 (PMC10246885; doi:10.34133/research.0157)
Supplement: Supplementary 1 — Figs. S1 to S6 Tables S1 to S3 [file research.0157.f1.docx]

Supplementary Materials


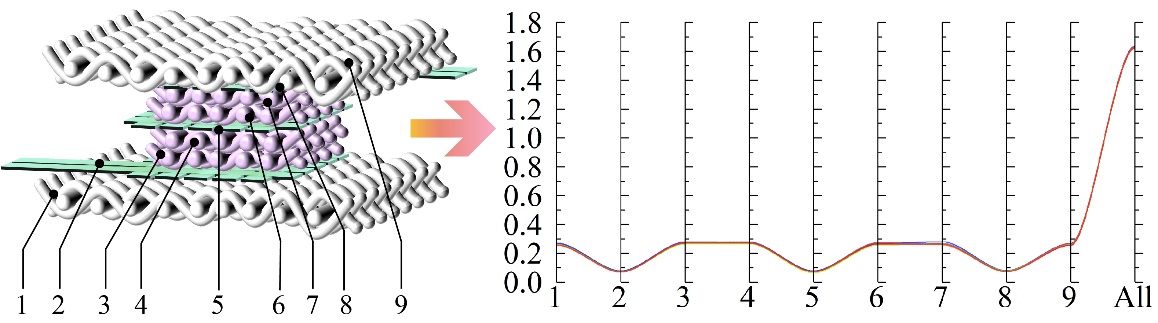


**Fig. S1.** The thickness of each fabric and the entire Gft sensor. (The lines show that 5 different sensors were measured)


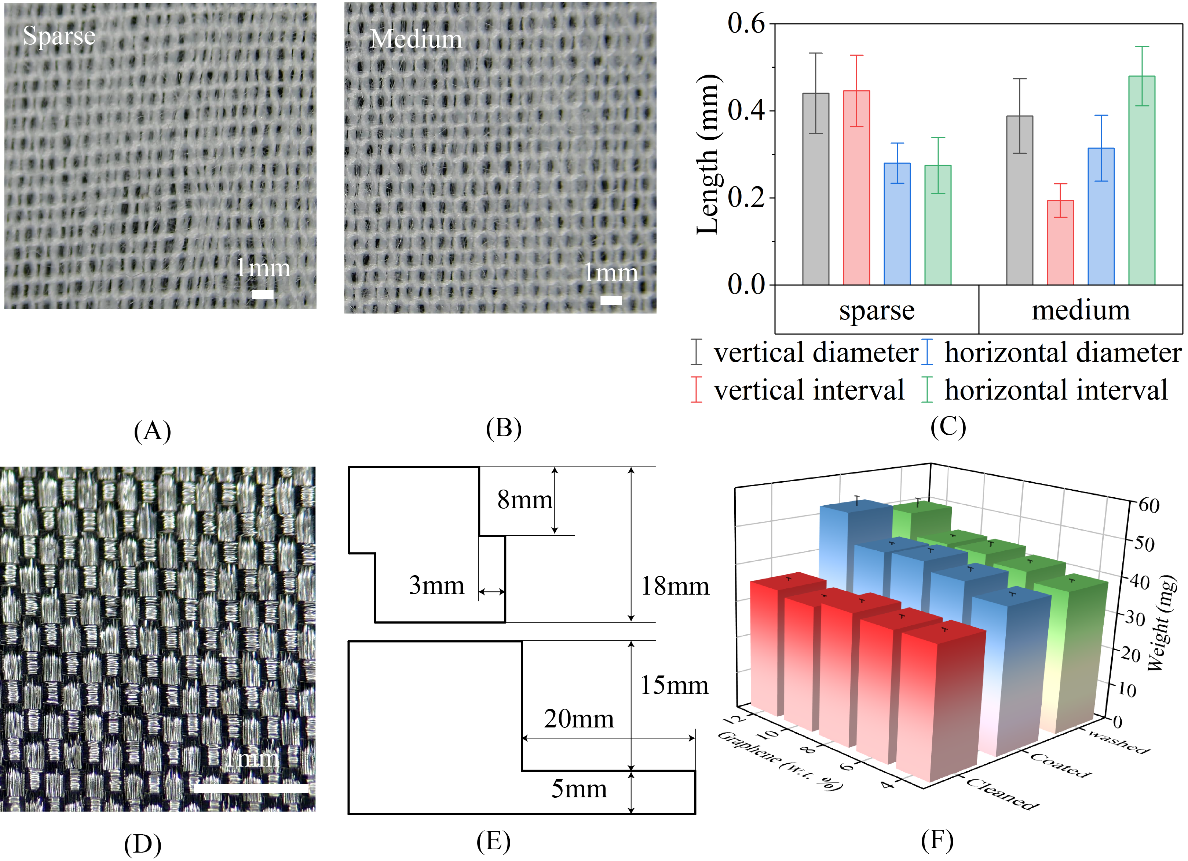


**Fig. S2.** The surface structure and quality change of sparse and medium-density polyester-cotton cloth after cleaning. (A-B) Microscopic Morphology of sparse and medium density polyester-cotton cloth under Optical Microscope. (C) Statistics of the surface structure of two kinds of polyester-cotton fabrics. (D) Microscopic Morphology of copper-nickel coated polyester fabric. (E) The shape of cut copper-nickel coated polyester fabric. (F) The quality change of sparse-density polyester-cotton after different concentration processes.


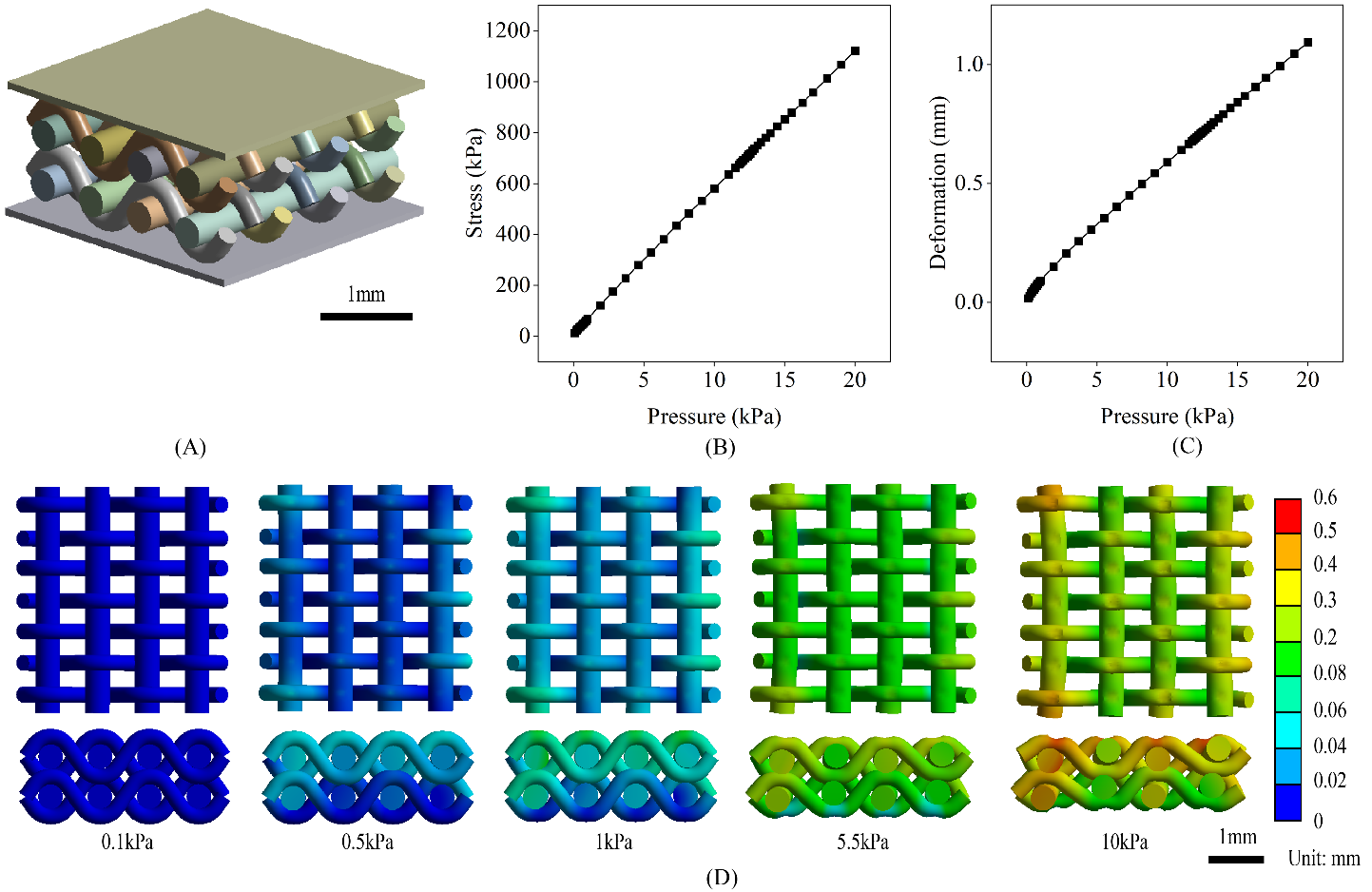


**Fig. S3.** Prototype and Results of Finite Element Analysis. (A) A simplified model of polyester-cotton weaving structure was established. (B) and (C) are the overall maximum stress and deformation of the prototype under different pressures, respectively. (D) Deformation distribution of the sensing layer under different pressures.


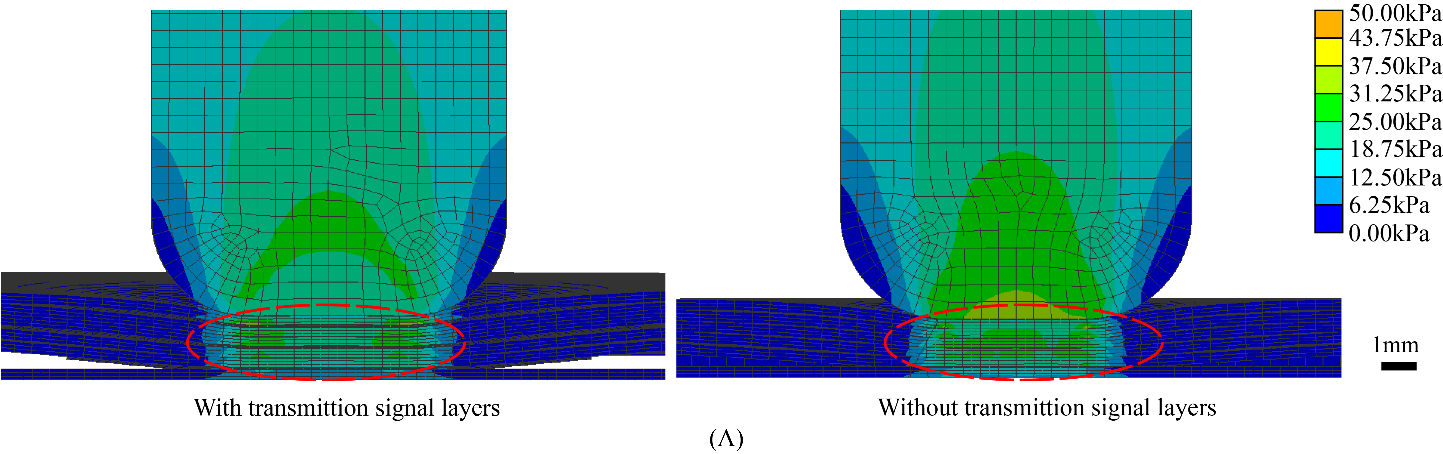


**Fig. S4.** FEM simulation output comparison without signal transmission layer. (A) The internal stress distribution of sensors with and without signal transmission layer under the same external force, respectively.


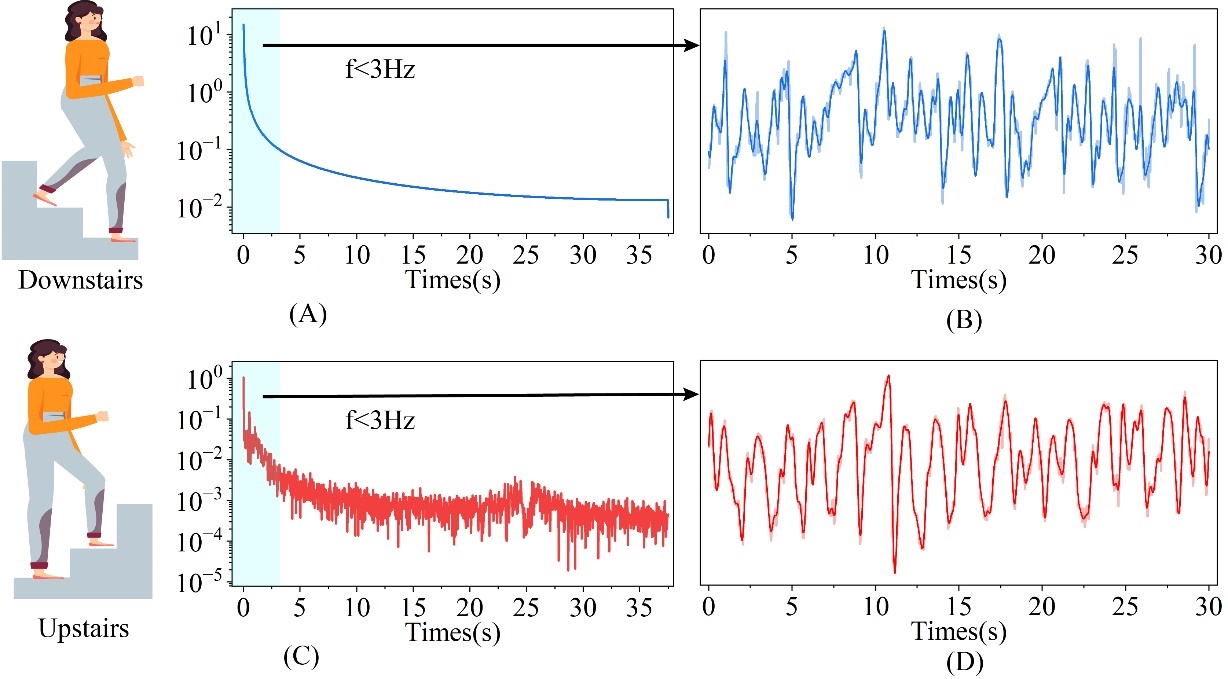


**Fig. S5.** Fast Fourier transform and filtering of cluttered waveforms. Frequency versus amplitude after FFT of sensor current responses as collected volunteers go downstairs (A) and upstairs (C). The original waveform (50% transparency) in the time domain and the waveform after 3Hz band-pass filtering (Opaque line) as collected volunteers go downstairs (B) and upstairs (D).


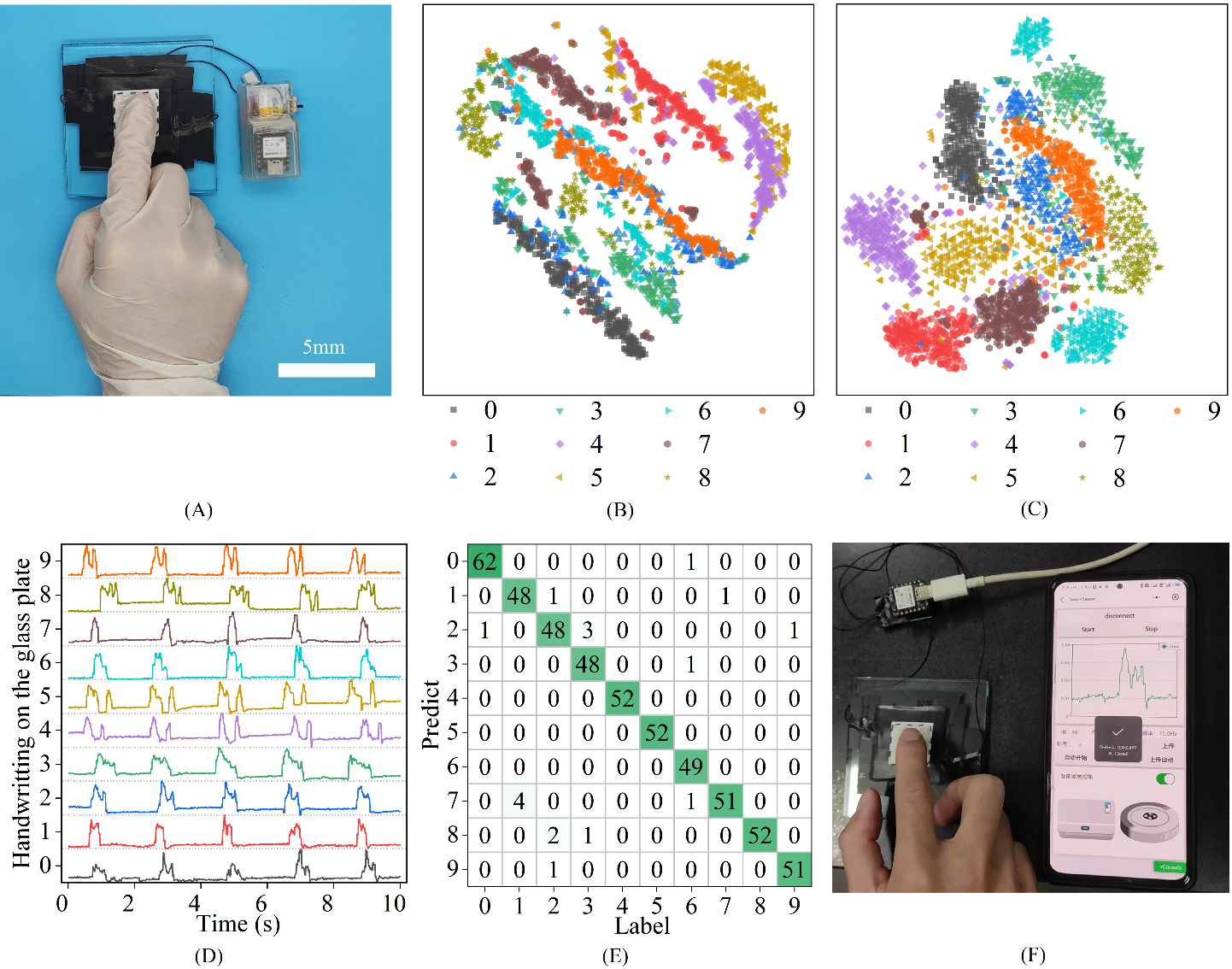


**Fig. S6.** Handwritten digits on the Gft sensor fixed on the glass plate. (A) Photograph of the Gft sensor on the glass plate. (B) and (C) are t-SNE dimensionality-reduced graphs of handwritten numeric data and features extracted by trained CNN, respectively. (D) Signal comparison of handwritten numbers on glass plates. (E) Confusion matrix of the recognition results of handwritten digits on glass plates test set. (F) Artificial IoT systems do cloud computing and return photos that identify the number "2".

**Table S1.** Manufacturing cost of one thousand Gft sensors.

| Material | Unit price | Required | Total price/yuan |
| --- | --- | --- | --- |
| Multi-layers graphene | 1.5 yuan/g | 4.6g | 6.9 |
| Sparse polyester-cotton textiles | 2.6875 yuan/m^2^ | 0.016m^2^ | 0.043 |
| Medium polyester-cotton textiles | 3.858 yuan/m^2^ | 0.032m^2^ | 0.123 |
| Metal coating fabric | 36.94 yuan/m^2^ | 0.012 m^2^ | 0.443 |
| total | | | 7.5($1.0885) |

**Table S2.** The comparison of the sensitivity (kPa^-1^) and linear pressure range (kPa) of this work with previous reports

| Sensitivity (kPa^-1^) | mechanical stability | Reference |
| --- | --- | --- |
| 0.042 (0-6 kPa), 0.33 (6-21 kPa), 0.037 kPa^-1^(21-110 kPa) | > 12000 cycles | [4] |
| 0.1124 (0-9 kPa), 0.0283 (9-37 kPa), 0.0021 (37-110 kPa) | > 100000cycles | [15] |
| 0.3213 (0.2-1 kPa), 0.0118 (5-20 kPa) | > 1000 cycles | [18] |
| 11.6 (0-0.25 kPa), 4.6 (0.25-3 kPa), 0.6(3-20 kPa) | > 5000 cycles | [20] |
| 0.21 (0-2 kPa), 0.0064 (2-10 kPa) | > 10000 cycles | [38] |
| 0.19 (0-0.5 kPa), 0.049 (0.5-3.5 kPa) | ~ 4000 cycles | [39] |
| 0.16 (0-10 kPa) | > 10000 cycles | [40] |
| 12.23 (0-13 kPa), 2 (13-52 kPa), 8.46 (52-80 kPa) | > 1000cycles | [41] |
| 0.008697 (0-4.5 kPa), 0.001275 (4.5-14 kPa), 0.00053 (14-140 kPa) | > 10000 cycles | [42] |
| 0.00136 (0-2 kPa), 0.000084 (2-10 kPa), | > 5000 cycles | [43] |
| 0.108 (0-2.5 kPa), 0.283 (4.5-6 kPa) | > 20000 cycles | [44] |
| 0.76 (0-1 kPa), 7.67 (1-2 kPa) | > 2000 cycles | [45] |
| 1.35 (5-600 kPa) | - | [46] |
| 7.168 (0-5 kPa), 0.808(5-10 kPa), 0.201 (10-20 kPa) | > 1000 cycles | [47] |
| 2.16 (0-155.485 kPa) | >1000000 cycles | This work |

**Table S3.** The layers and parameters of Gft-net.

| Layer (type) | Output shape | Parameters |
| --- | --- | --- |
| Conv1d-1 | [Batch size, 16, 141] | 176 |
| BatchNorm1d-2 | [Batch size, 16, 141] | 32 |
| MaxPool1d-3 | [Batch size, 16, 70] | 0 |
| Dropout-4 | [Batch size, 16, 70] | 0 |
| ReLU-5 | [Batch size, 16, 70] | 0 |
| Conv1d-6 | [Batch size, 32, 61] | 5,152 |
| BatchNorm1d-7 | [Batch size, 32, 61] | 64 |
| MaxPool1d-8 | [Batch size, 32, 20] | 0 |
| Dropout-9 | [Batch size, 32, 20] | 0 |
| ReLU-10 | [Batch size, 32, 20] | 0 |
| Conv1d-11 | [Batch size, 64, 18] | 6,208 |
| BatchNorm1d-12 | [Batch size, 64, 18] | 128 |
| MaxPool1d-13 | [Batch size, 64, 6] | 0 |
| Dropout-14 | [Batch size, 64, 6] | 0 |
| ReLU-15 | [Batch size, 64, 6] | 0 |
| Linear-16 | [Batch size, 256] | 98,560 |
| BatchNorm1d-17 | [Batch size, 256] | 512 |
| Dropout-18 | [Batch size, 256] | 0 |
| ReLU-19 | [Batch size, 256] | 0 |
| Linear-20 | [Batch size, Class number] | 2,570 |
